# Analysis of the day 3 transfer strategy for POSEIDON patients

**DOI:** 10.5935/1518-0557.20240111

**Published:** 2025

**Authors:** Jakub Wyroba, Joanna Kochan

**Affiliations:** 1 Malopolski Institute of Fertility Diagnostics and Treatment - krakOvi, Krakow, Poland; 2 Fertility Disorders Clinic, Andrzej Frycz Modrzewski Krakow University, Krakow, Poland; 3 Department of Animal Reproduction, Anathomy and Genomics, University of Agriculture, Krakow, Poland

**Keywords:** POSEIDON, low responders, embryos, ICSI, ET day 3, ET

## Abstract

**Objective:**

The aim of the study was to analyze the effectiveness of the day 3 ET strategy, and the morphology of the transferred embryos, in patients from POSEIDON and non-POSEIDON groups.

**Methods:**

600 cycles of patients meeting the POSEIDON criteria and 600 non-POSEIDON cycles were analyzed to determine the proportion of cycles with an ET on days 3 or 5, or FET. Then we reviewed 330 day 3 ETs to compared the developmental stage, morphology, zona pellucida thickness and implantation potential of embryos transferred on day 3 from POSEIDON and non-POSEIDON patients.

**Results:**

Most cycles of POSEIDON patients end with ET on day 3 (42%) or without transfer (37%). In contrast, most cycle of non-POSEIDON patients end with FET (44%) and just 9% is canceled. The lowest percentage of embryos at the morula stage was recorded in POSEIDON groups III (10%) and IV (9%). The average number of cells in embryos was comparable in all groups. The largest percentage of top-quality embryos (grade A) were in POSEIDON group I (47%) .The highest implantation potential were observed in the non-POSEIDON group <35Y (28%), and in POSEIDON groups I (28%) and III (26%). The highest incidence of miscarriage was recorded in all POSEIDON and non-POSEIDON groups that included patients who were ≥35 years of age.

**Conclusions:**

The day 3 ET strategy still seems optimal for POSEIDON patients. The prognosis depends on which Poseidon group the patient is in. The best prognosis is for group I and the worst for group IV.

## INTRODUCTION

Optimal standards of treatment management for IVF patients with a poor ovarian response (POR) have been sought for many years. In 2016, the POSEIDON Group, comprised of specialists in reproductive endocrinology and reproductive medicine, proposed more detailed definitions of POR than those in the Bologna criteria (Ferraretti *et al.*, 2011), including the creation of 4 POSEIDON (Patient-Oriented Strategies Encompassing Individualized Oocyte Number) groups for low prognosis patients, based on age, AMH level or oocyte number (Poseidon Group, 2016). Since then, a number of therapeutic paths have been proposed for specific groups of POSEIDON patients undergoing IVF (Giannelou *et al.*, 2020; Haahr *et al.*, 2019; Sunkara *et al.*, 2020). However, most analyses have focused on ovarian stimulation protocols, as retrieving a satisfactory number of oocytes is the basis for further IVF procedures. However, there are far fewer publications regarding the optimization of subsequent stages of the IVF procedure for these patients.

One of the most important clinical stages of IVF, apart from ovarian stimulation and obtaining oocytes, is the embryo transfer (ET). Moreover, waiting for the embryo transfer and then for its result are some of the most stressful times for couples during the entire IVF process (Csemiczky *et al.*, 2000). Deciding whether to transfer on day 3 or 5 is extremely important for patients with POR, from whom we obtain several oocytes and usually just one or two embryos. Very often, POR (POSEIDON) patients have no opportunity for a second transfer from the cycle, using cryopreserved embryos (FET), because there are no additional embryos for vitrification, which generates an additional mental burden. Therefore, specific communication between the physician and the couple regarding their cycle is very important.

The couple should be aware that their inclusion in a POSEIDON group could reduce their chance of IVF success. Patients should be prepared for the possibility of ET on either day 3 or day 5, and not be surprised by a seemingly spontaneous decision by the physician and embryologist. A range of ET strategies have been proposed for patients with poor ovarian response, including transfer of cleavage-stage embryos on day 3 or even on day 2, or transfer of a blastocyst on day 5, while others suggest the “freeze all” strategy (Berkkanoglu *et al.*, 2017; Hu *et al.*, 2023; Shahine *et al.*, 2011). Because in vitro conditions are never as ideal as in vivo conditions for embryo development, it would seem logical to shorten the time the embryo stays in culture. On the other hand, by extending the culture period, we have more information about the embryo’s development and can make a more informed selection (Garbhini *et al.*, 2023). On day 5, we can assess more morphokinetic parameters such as blastocyst expansion, as well as the quality of the ICM (inner cell mass) and TE (trophoectoderm) cells (Gardner *et al.*, 2000). In addition, blastocysts can be biopsied for PGT-A (preimplantation genetic testing for aneuploidy), allowing the selection of euploid embryos for ET. When we transfer embryos on day 3, we have much less information about the quality of the embryo, as we can only evaluate the embryos in terms of the number and symmetry of blastomeres and the degree of cytoplasmic fragmentation. However, if we maintain single embryos in culture until day 5, we risk ET failure due to the lack of a blastocyst, which causes the couple great stress, and can lead to their reluctance to undergo further IVF cycles. Moreover, in POSEIDON patients, especially those in groups III and IV, we do not often have the option of selecting embryos, and so even poor quality embryos are transferred, reducing the effectiveness of the ET.

Therefore, the aim of the study was to analyze the effectiveness of the day 3 ET strategy, and the morphology of the transferred embryos, in patients from POSEIDON and non-POSEIDON groups.

## MATERIALS AND METHODS

This was a retrospective study of patients who underwent intracytoplasmic sperm injection (ICSI) and single embryo transfer on day 3 in the Krakovi Clinic in Kraków (Poland) from 2021-2024. The research was carried out in accordance with the guidelines of the local bioethics committee (KBKA/7/O/2024).

### Study design

**Figure 1:**
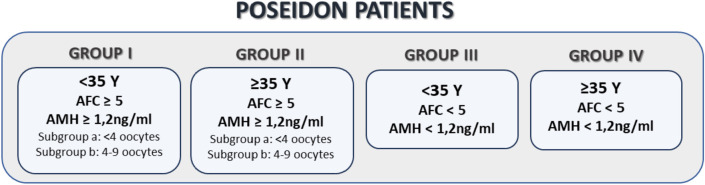
POSEIDON criteria of low prognosis patients.

In the first stage of the study, total of 600 cycles of patients meeting the POSEIDON criteria according to Poseidon Group (2016) (Fig. 1) and 600 non-POSEIDON cycles were analyzed to determine the proportion of cycles with an ET on days 3 or 5, or FET in each groups. In our study, we did not divide POSEIDON groups I and II into subgroups.

In the second stage, we compared the developmental stage, morphology and implantation potential of embryos transferred on day 3 from POSEIDON patients and from non-POSEIDON patients. Embryos were evaluated immediately before ET.

The following embryo characteristics were analyzed;

- percentage of morulae on day 3

- average number of cells in the embryo (excluding morulae stage)

- cytoplasmic fragmentation (grade A, B)

- average thickness of the zona pellucida (ZP)

- percentage of embryos with extremely thin (≤ 12 µm) and extremely thick (≥ 17 µm) ZP

-implantation potential (clinical pregnancy and ongoing pregnancy rate)

### Clinical protocols

Patients were treated using either the long agonist protocol or short antagonist protocol. The type of protocol used depended on the level of AMH and the overall risk of hyperstimulation.

***Long agonist protocol:*** Starting 1 week before the expected menses (cycle day 18‒23), patients received the GnRH agonist, triptorelin (Decapeptyl *Ferring Pharmaceuticals*, 1 mg/d, sc). After successful pituitary *downregulation* (when the serum estradiol (E2) levels were < 40 pg/mL), ovarian stimulation was commenced with a fixed daily dose of 150-300 IU recombinant follitropin alfa (rFSH, sc) with or without an additional 75‒150 IU menotropin (hMG).

***Antagonist protocol:*** A GnRH antagonist Cetrorelix (Cetrotide *Merck Europe* , 0.25 mg/d, sc or Ganirelix *Gedeon Richter* 0.25 mg/d), was administered, commencing when the largest follicle reached a diameter of 14 mm. rFSH/hMG was initiated on day 2-4 of the cycle.

The agonist and antagonist protocols were continued up to and including the day of human chorionic gonadotropin (hCG) administration, which was when the leading follicle reached a diameter of 18 mm or more and at least three follicles reached a diameter of 17 mm or more. rFSH was then stopped, and a single sc bolus of 10,000 IU hCG (Eutrig - *Samarth Life Sciences*) or 6,500 IU rhCG (Ovitrelle - *Merck*) was administered 36 h before the planned time of oocyte retrieval. When there was a risk of OHSS in an antagonist cycle, the trigger was a single sc bolus of triptorelin 2mg, and a freeze-all policy was applied. All follicles 12 mm or larger were aspirated. Subsequently, the oocytes were inseminated via ICSI, and a single embryo was transferred 3 days later. Luteal support in the form of intravaginal progesterone (Cyclogest - *Gedeon Richter*, 400 mg twice a day) was administered starting from the day after oocyte retrieval until a serum pregnancy (b-hCG) test was performed 17 days later.

### Ovarian stimulation monitoring in ICSI

Baseline blood sampling and transvaginal sonography (TVS) was performed on day 2 or 3 of the treatment cycle for all patients. Monitoring of response during the treatment cycle consisted of TVS and blood sampling for hormonal analysis on cycle days: 2-3 (E_2_, FSH, LH); 5-6 (E_2_); 8-9 (E_2_); and day of hCG administration (E_2_, P_4_). Additional TVS monitoring was performed as clinically indicated.

### Clinical outcome measures

Clinical pregnancy was defined by the ultrasound confirmation of an intrauterine gestational sac after 8 weeks of gestation with visible fetal cardiac activity. Ongoing pregnancy was defined when the pregnancy had completed over 12 weeks of gestation with visible fetal cardiac activity.

### Laboratory protocols

Oocyte-cumulus complexes (COCs) were identified using a stereoscopic microscope and then washed and incubated (approx. 3 h) in Washing medium (Gynemed, Germany) under a 6.0% CO_2_, 5.0% O_2_ atmosphere. After incubation, oocytes were denudet using hyaluronidase and mechanical pipetting. Only oocytes in metaphase II with a first polar body were used for further procedures. Intracytoplasmic sperm injection (ICSI) was performed using an RI Integra 3 micromanipulator (Research Instruments, Germany) following the standard technique. Embryos were cultured in SAGE^®^ medium (Origio, Denmark) under an atmosphere of 6.0% CO_2_, 5.0% O_2_ and balance nitrogen at 37°C. Embryo development was assessed every day. On day 3, immediately before ET, the embryo was assessed based on the number of cells and the degree of cytoplasmic fragmentation (A, B). During the evaluation, a photo of the embryo was taken at 40x magnification for later determination of zona pellucida thickness (ZPT). ZPT was measured at four points to determine the average thickness, using MultiScan^®^ software.

### Statistical analysis

Non-parametric data, such as differences in the percentage values between groups, were assessed by the chi-squared test. Parametric data were expressed as means±SD and compared by two-way ANOVA. Differences were considered significant when the *p*-value was ≤0.05. The statistical analysis was performed using PQStat 1.6.2 (PQStat Soft, Poznan, Poland).

## RESULTS

**Figure 2:**
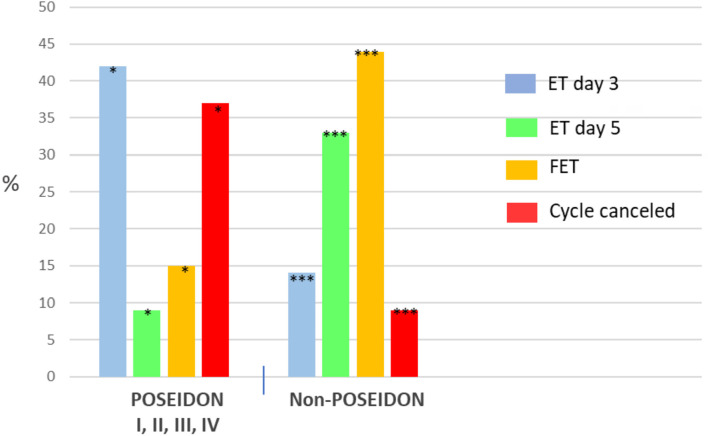
Embryo transfer strategy for POSEIDON and non-POSEIDON patients.

**Figure 3:**
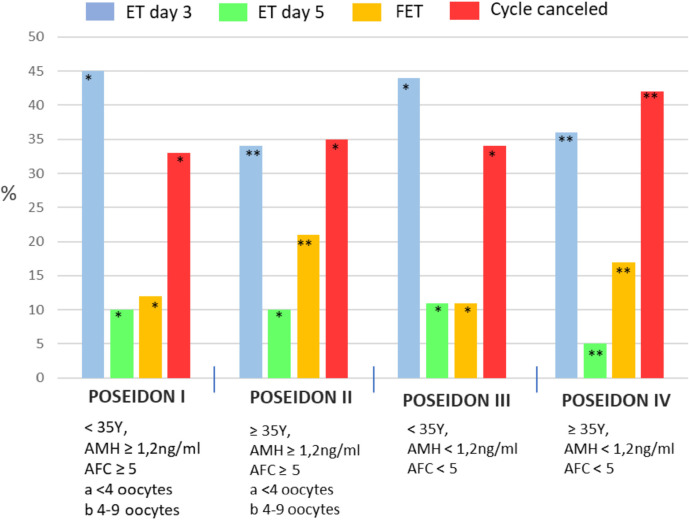
Embryo transfer strategy for each group of POSEIDON patients.

In the first stage of the study, 600 cycles of POSEIDON and 600 of non-POSEIDON patients were analyzed to determine the percentage of ET on day 3 or 5 and FET (Fig. 2, Fig. 3). In our Center, most cycles of POSEIDON patients end with ET on day 3 (42%) or without transfer (37%) due to the lack of oocytes or embryos. In contrast, most cycle of non-POSEIDON patients end with FET (44%) and just 9% is canceled. Of all ETs on day 3 in our Center during the study period, most (45%) were performed in POSEIDON I group (Fig. 3). The basic characteristics of each POSEIDON and non-POSEIDON groups with ET on day 3 are presented in Table 1.

**Table 1:** Baseline characteristics of patients with ET on day 3.

Parameters	Total	POSEIDON				NON POSEIDON
		G I n (%)	G II n (%)	GIII n (%)	G IV n (%)	
No of cycles n (%)	330	50 (15%)	40 (12%)	63 (19%)	89 (27%)	88 (27%)
Age (years) mean±SD	35.2±4.7	31.2±2.6	37.5±2.2	30.4±2.5	38.8±2.4	35.8±.4.5
BMI kg/m^2^ mean±SD	23.1±4	22.8±4	23.3±5	22.3±4	23.3±4	22.9±3
AMH ng/ml mean±SD	1.8±1.6	2.8±1.3	1.7±0.7	0.66±0.5	0.55±0.5	3.1±2
No of oocytes mean±SD	4±2.1	3±1.16	3±1.17	2±1	2±1.2	11±3
No of cleaved embryos	2.3±0.8	2.7±0.4	2.4±0.3	1.7±0.5	1,4±0.3	5±2.1
Number of remaining blastocysts after ET on day 3 mean±SD	0.7±0.3	0.5±0.1	0.4±0.09	0.2±0.1	0.2±0.07	1.4±0.9

ET - embryo transfer, AMH - Anti-Mullerian hormone, BMI - the body-mass index is the weight in kilograms divided by the square of the height in meters.

### Embryo stage and morphology at day 3

Table 2 presents the analysis of the morphology and developmental stage of embryos on day 3 immediately before ET for patients who did and did not meet the POSEIDON criteria. The lowest percentage of embryos at the morula stage was recorded in POSEIDON groups III (10%) and IV (9%), which are characterized by AMH<1.2 ng/ml, regardless of age. However, in the POSEIDON I, II and non-POSEIDON groups, the morula rate was comparable, at 15-16%. The average number of cells in embryos (without morula analysis) was comparable in all groups. The largest percentage of top-quality embryos (grade A, Fig. 3) were in POSEIDON group I (47%) and in the non-POSEIDON <35y group (41%).

**Table 2:** Characteristics of embryos transferred on day 3.

Patient groups	Number of cells in the embryo mean±SD	Morula stage n (%)	Embryo quality, grade A n (%)
**POSEIDON I** n=50 <35Y, ≥1.2 ng/ml AMH	8.8±0.5	8 (16%)^a^	23 (47%)^a^
**POSEIDON II** n=40 ≥35Y, ≥1.2 ng/ml AMH	8±0.3	6 (15%)^a^	16 (39)^a^
**POSEIDON III** n=63 <35Y, <1.2 ng/ml AMH	8.1±0.7	6 (10%)^b^	16 (25)^b^
**POSEIDON IV** n=89 ≥35Y, <1.2 ng/ml AMH	8.1±0.5	8 (9%)^b^	24 (27)^b^
**Non-POSEIDON** <35y, n=39	8.2±0.2	6 (15%)^a^	16 (41%)^a^
**Non-POSEIDON** ≥35y, n=49	8.1±0.7	7 (15%)^a^	15 (30%)^b^
Total n=330	8.1±0.5	36 (12%)	107 (32%)

a:b values with different superscripts within the same column differ significantly (*p*<0.05).

### Thickness of zona pellucida

**Figure 4:**
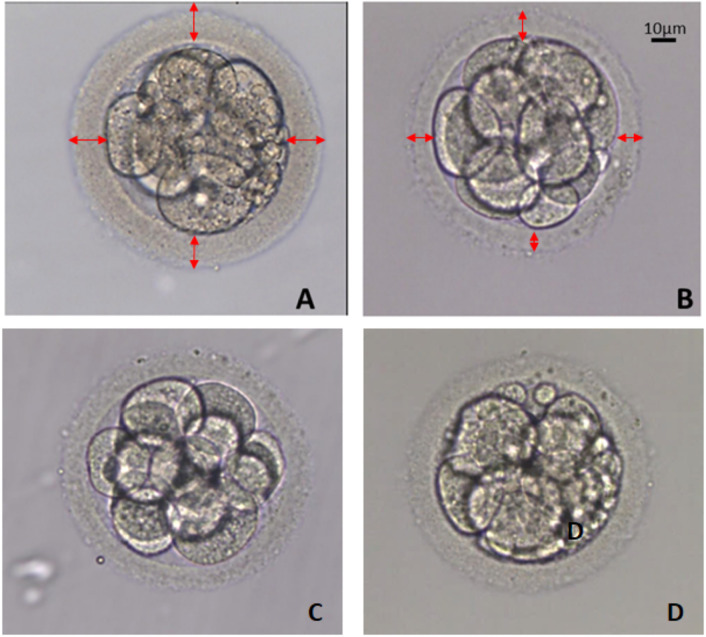
Morphology of embryos on day 3. A. Embryo with extremely thick ZP (20µm), B- Embryo with extremely thin ZP (11µm). C- Embryo grade A. D- Embryo grade B.

The range of zona thickness was 9-22 µm (Table 3). We did not observe any significant differences in the mean thickness of the ZP between the study groups. However, we did observe that embryos from the POSEIDON and non-POSEIDON groups ≥35y were more likely to have an extremely thin ZP than those from other groups < 35y (*p*<0.05, *p*<0.001; Fig 4.) We also observed more embryos with a thick ZP in the POSEIDON I, III and non-POSEIDON <35y groups compared to those from the older patients in the POSEIDON II (*p*<0.05), POSEIDON IV and non-POSEIDON groups (*p*<0.001). No effect of AMH level on zona thickness was detected between groups.

**Table 3:** Thickness of the zona pellucida (ZPT) on day 3.

Patient groups	ZPT (µm) mean±SD	ZP thin (≤12µm) n (%)	ZP thick (≥17 µm) n (%)
**POSEIDON I** n=50 <35Y, ≥1.2 ng/ml AMH	15.5±2.2	10 (21%)^b^	14 (28%)^a^
**POSEIDON II** n=40 ≥35Y, ≥1.2 ng/ml AMH	15.7±2.0	12 (30%)^a^	6 (14%)^c^
**POSEIDON III** n=63 <35Y, <1.2 ng/ml AMH	15.4±1.9	10 (17%)^c^	19 (30%)^a^
**POSEIDON IV** n=89 ≥35Y, <1.2 ng/ml AMH	14.1±1.9	30 (34%)^a^	9 (10%)^c^
**Non-POSEIDON** <35Y, n=39	15.6±2.1	7 (18%)^c^	13 (33%)^a^
**Non-POSEIDON** ≥35Y, n=49	14.7±2.0	14 (27%)^a^	8 (17 %)^c^
Total n=330	14.9±2.0	81 (25%)	69 (21%)

a:b ,

b:c values with different superscripts within the same column differ significantly (*p*<0.05)

a:c values with different superscripts within the same column differ highly significantly (*p*<0.001).

### Implantation potential

Following a total of 330 day 3 ETs, we achieved a clinical pregnancy rate of 21%, with an ongoing pregnancy rate of 18% (Table 4). The highest clinical pregnancy rate was noticed in the non-POSEIDON group <35y (28%), and in POSEIDON groups I (28%) and III (26%). Embryos from Poseidon groups II and IV, which included patients aged 35 years or older, had the lowest implantation potential (17% and 13%, respectively). The highest incidence of miscarriage was recorded in all POSEIDON and non-POSEIDON groups that included patients who were ≥35 years of age.

**Table 4:** Day 3 ET outcomes in POSEIDON and non-POSEIDON groups.

Patient groups	Clinical pregnancy n (%)	Ongoing pregnancy n (%)
**POSEIDON I** n=50 <35Y, ≥1.2ng/ml AMH	13 (28%)^a^	13 (26%)^a^
**POSEIDON II** n=40 ≥35Y, ≥1.2ng/ml AMH	7 (17%)^b^	6 (15%)^b^
**POSEIDON III** n=63 <35Y, <1.2ng/ml AMH	16 (26%)^a^	15 (24%)^a^
**POSEIDON IV** n=89 ≥35Y, <1.2ng/ml AMH	12 (13%)^b^	10 (11%)^b^
**Non-POSEIDON** <35Y, n=39	11 (28%)^a^	10 (25%)^a^
**Non- POSEIDON** ≥35Y, n=49	9 (18%)^b^	7 (14%)^b^
Total n=330	69 (21%)	61 (18%)

a:b values with different superscripts within the same column differ significantly (*p*<0.05).

## DISCUSSION

Poor ovarian response occurs in 9-25% of all ART cycles (Jenkins *et al.*, 1991; Surrey & Schoolcraft, 2000). In our Center, which specializes in the treatment of patients with a poor ovarian response, as many as 33% of all patients meet the POSEIDON criteria. Although there are a number of embryo transfer strategies for POR patients, ET on cycle day 3 remains the most popular (Chinta *et al.*, 2021; Lebovitz *et al.*, 2022; Nichi *et al.*, 2011). Similarly, in our Center, most embryos (42%) from POR patients are transferred on day 3. The FET strategy (15%) usually assumes the assessment of ploidy via PGT-A, and is most often proposed for the POSEIDON II and IV groups, which include older patients (≥35 years of age). Although embryo transfer on day 5 (9%) allows for better embryo selection, it is usually used in POSEIDON patients with a larger number of oocytes and embryos. Extending embryo culture to day 5 with a low number of oocytes could result in the failure to obtain a blastocyst and in the need for cancellation of the ET. Taking into account the very low rates of obtaining additional blastocysts after ET on day 3 blastocysts per cycle (mean 0.7; Table 1), the strategy adopted here seems to be optimal. Despite minimizing this risk, ET is still cancelled in 37% of POSEIDON patients in our Centre, due to lack of oocytes or embryos, similar to reports by other authors (Berkkanoglu *et al.*, 2017; Klinkert *et al.*, 2004; Lamazou *et al.*, 2012). An interesting proposition is sequential (two-step) day 3/day 5 frozen-thawed embryo transfer in POR patients, which has been shown to be associated with a higher live birth rate compared with the traditional double cleavage-stage ET (44.2% *vs*. 34.3%), but this strategy requires even more careful evaluation (Hu *et al.*, 2023).

In our study, we analyzed the morphology of embryos transferred on day 3 in 4 groups of POSEIDON patients, concluding that problems with the number of oocytes may also be related to their developmental capacity and to the embryo morphology. While it has been reported that POR patients can produce top quality embryos (Lebovitz *et al.*, 2022; Nichi *et al.*, 2011) embryo morphology was not compared between POSEIDON groups. In our study, the largest proportion of top quality embryos (47%), as well as embryos at an advanced stage of development on day 3 (morula-16%) were observed in POSEIDON I patients, who were young with normal AMH levels, and in whom a poor ovarian response was unexpected. The poorest quality embryos were observed in the POSEIDON III and IV groups, with AMH <1.2 ng/ml, and in the non-POSEIDON >35Y group. In these groups, the fewest oocytes and embryos were obtained, and there were limited opportunities to select embryos for ET. Therefore, even poor quality embryos were transferred.

We also investigated the thickness of the zona pellucida in POSEIDON patients’ embryos. Although zona thickness and structural changes have been studied extensively, there is still no clear consensus on the relationship between the ZPT and the patient’s age. According to some authors, there is strong evidence that the thickness of human ZP is not influenced by the patient’s age (Balakier *et al.*, 2012), while others have reported either positive (Nawroth *et al.*, 2001; Shiloh & Dirnfeld, 2001) or negative (Gabrielsen *et al.*, 2000; Garside *et al.*, 1997; Sun *et al.*, 2005) correlations. It is possible that this discrepancy in results between studies is due to the inclusion of patients from different age groups and the use of different methods of obtaining the embryo (IVF vs. ICSI). According to Balakier *et al.* (2012) embryos generated from routine IVF had significantly thinner ZP than those from ICSI procedure. There have also been several studies on the influence of hormonal status on ZPT (Balakier *et al.*, 2012; Shiloh & Dirnfeld, 2001; Shulman *et al.*, 1993) with basal level of FSH and high E2 levels shown to have no significant effect (Balakier *et al.*, 2012). However, there have been no studies on the influence of ovarian reserve on ZPT. In our study, although no differences were detected between the study groups in the average thickness of the ZP, we observed a higher percentage of embryos with a very thick ZP in the POSEIDON I, III and non-POSEIDON <35y groups compared to embryos from older patients in the POSEIDON II, IV and non-POSEIDON groups. This suggests a relationship with the age of the patients and not the ovary’s response to stimulation. It has been well recognized that embryo morphology is a significant predictor of implantation rate (Awadalla *et al.*, 2021; Balaban *et al.*, 2006; Shulman *et al.*, 1993). However, in the case of POR patients, there is often no opportunity to choose the best quality embryo because we only have one embryo, and so even poor quality embryos are transferred to the uterus. Based on a multicenter cohort study, the cumulative delivery rate per IVF/ICSI cycle of is on average 50% lower in POSEIDON patients than in normal responders, and this varies across POSEIDON groups (Esteves *et al.*, 2021). In our study, the highest clinical pregnancy rates were observed in the non-POSEIDON<35y group (28%) and in POSEIDON groups I (28%) and III (26%) (Table 4). Embryos from POSEIDON groups II (17%) and IV (13%) had the lowest implantation potential. Patients aged 35 years or older had the highest miscarriage rate, in both the POSEIDON and non-POSEIDON groups. Our embryo implantation results on day 3 in the POSEIDON groups were comparable to those published by Chinta *et al.* (2021), and significantly better than those of Eftekhar *et al.* (2018).

A limitation of our research is that the POSEIDON groups are unequal. Despite the large initial number of POR patients, dividing them into four categories results in groups with disproportionate numbers of patients. The smaller groups, I and II, are particularly problematic. We note that other authors have had a similar problem, with group II being the smallest (Chinta *et al.*, 2021; Eftekhar *et al.*, 2018). Also, the POSEIDON groups in the cohort studies of Esteves *et al.* (2021) are disproportionate in size, although there it was group III that was the smallest. The advantage of our research is that it was performed in one Center, so the laboratory conditions and all procedures were identical for all patients and all embryos. Further, the POSEIDON classification has only been in use for 8 years, so any publication that provides detailed information regarding the specificity of individual groups of POSEIDON patients and the relationships between them is important and may help in developing a treatment management strategy.

The day 3 ET strategy still seems optimal for POSEIDON patients, especially those with low oocyte and embryo counts. However, POSEIDON patients should be prepared for an embryo transfer on day 3, and be aware of its advantages and disadvantages. The prognosis depends on which Poseidon group the patient is in. The best prognosis is for group I and the worst for group IV.
